# Indications and Surgical Techniques for Repeat Corneal Transplantation in Eastern China: A Twelve-Year Study

**DOI:** 10.1155/2021/5514004

**Published:** 2021-09-30

**Authors:** Xichen Wan, Wang Yao, Songjiao Zhao, Jianjiang Xu, Qihua Le

**Affiliations:** ^1^Department of Ophthalmology Eye Ear Nose and Throat Hospital, Fudan University, Shanghai, China; ^2^Research Centre Eye Ear Nose and Throat Hospital, Fudan University, Shanghai, China; ^3^Myopia Key Laboratory of Ministry of Health Eye Ear Nose and Throat Hospital, Fudan University, Shanghai, China

## Abstract

**Purpose:**

To analyze the indications and surgical procedures for repeat keratoplasty in eastern China from 2008 to 2019.

**Methods:**

This retrospective descriptive study included 418 eyes of 411 patients who underwent no less than 2 keratoplasties at the Eye, Ear, Nose and Throat Hospital of Fudan University from 2008 to 2019. Medical charts were reviewed. The primary indications for repeat keratoplasty, the reasons for regrafting, and the surgical techniques used in the treatment were collected and analyzed.

**Results:**

Among 418 eyes, 337 eyes (80.6%) had one repeat keratoplasty, and 81 eyes (19.4%) had multiple repeat keratoplasties (≥2 repeat keratoplasties). The median interval between the initial keratoplasty and the first repeat keratoplasty was 25 months, and that between two keratoplasties after the first repeat keratoplasty was 27.5 months. Infectious keratitis was the leading primary indication for single repeat keratoplasty (80 cases, 23.7%) and multiple repeat keratoplasties (19 cases, 23.5%). The second most common primary indication was bullous keratopathy for single repeat keratoplasty (49 eyes, 14.5%) and chemical injury for multiple repeat keratoplasties (14 eyes, 17.3%). The main reason for regrafting was allograft rejection (262 cases, 49.3%), followed by endothelial dysfunction (92 cases, 17.3%), and for vision improvement after tectonic keratoplasty (60 cases, 11.3%). Penetrating keratoplasty (PKP) was the major technique used in repeat keratoplasty (447 cases, 84.2%). However, Descemet stripping endothelial keratoplasty was more frequently used than PKP (72.4% vs. 27.6%, *P* < 0.001) in the treatment of failed endothelial keratoplasty.

**Conclusion:**

Infectious keratitis was still the leading cause of repeat keratoplasty in eastern China. Although PKP remains the major technique of repeat keratoplasty, the application of customized lamellar keratoplasty has greatly expanded in the last decade. Cautious selection of indications, surgical techniques, and timing for surgery is crucial for a good prognosis after repeat keratoplasty.

## 1. Introduction

With the widespread use of keratoplasty in the treatment of corneal blindness and increasing life expectancy, regraft has been one of the most common indications for keratoplasty in recent decades [1–5]. Repeat keratoplasty is technically more challenging. Even with careful preoperative evaluation and postoperative management, regraft still has a higher rate of graft failure, shorter survival time, and worse prognosis [6].

It has been reported that primary indications have an important impact on the prognosis of repeat keratoplasty. The leading primary indication greatly varies among different countries [4, 7–9], and it was reported to be pseudophakic bullous keratopathy in developed countries [4, 6] and vascularized corneal scarring in developing countries [7]. Moreover, the reasons for graft failure after the first keratoplasty, such as endothelial dysfunction [10] and allograft rejection [11], were also significantly correlated with the long-term outcome of the regraft.

Penetrating keratoplasty (PKP) remains the main surgical technique of repeat keratoplasty worldwide [3]. However, with the development of various lamellar keratoplasties and endothelial keratoplasties in recent decades, its use has gradually expanded in repeat keratoplasty. [12–14] Renovations of surgical techniques provide more customized options in repeat keratoplasty and to a certain extent improve surgical outcomes [13, 15]. To the best of our knowledge, although several investigations on repeat corneal transplantation have been published [4, 9, 16], the studies on clinical characteristics of repeat keratoplasty in China was rather limited, especially on multiple keratoplasties. Therefore, we performed this retrospective study to investigate the primary indications for and surgical techniques of repeat keratoplasty and to explore the reasons for regrafting.

## 2. Materials and Methods

### 2.1. Patients

All cases of repeat keratoplasties that were performed at the Eye, Ear, Nose and Throat Hospital of Fudan University from 1 January 2008 to 31 December 2019 were included. Eyes with 2 or more repeat keratoplasties were classified as having multiple repeat keratoplasties. The demographic data, primary indications for the first keratoplasty, reasons for single repeat keratoplasty or multiple repeat keratoplasties, and surgical procedures of each keratoplasty were collected. This study conformed to the Declaration of Helsinki and was approved by the Ethics Committee of the Eye, Ear, Nose and Throat Hospital of Fudan University.

### 2.2. Primary Indications for Keratoplasty and Reasons for Regraft

Primary indications for keratoplasty were divided into three categories: acquired nontraumatic, congenital abnormalities, and acquired traumatic [17]. Acquired nontraumatic included infectious keratitis, bullous keratopathy (except those caused by Fuchs dystrophy), corneal scarring, keratoconus, noninfectious keratitis, and corneal degeneration. Infectious keratitis included viral keratitis, fungal keratitis, bacterial keratitis, Acanthamoeba keratitis, and infection with unidentified pathogens. Corneal dystrophy, including Fuchs' dystrophy, was classified as congenital abnormalities because of its hereditary component. The detailed classification of each category is shown in Table 1.

The reasons for regrafting are listed in Table 2. Allograft rejection was diagnosed based on the appearance of graft edema, the rejection line, new keratic precipitates, or aqueous cells. The presence of irreversibly decreasing endothelial cell density and loss of graft clarity was defined as endothelial dysfunction [6, 18]. Persistent epithelial defect was determined when the presence of ≥2 mm patchy graft epithelial defect lasted longer than 2 weeks and did not respond to intensive tear substitutes, therapeutic contact lens, and tarsorrhaphy [19]. Irreversibly diffuse graft edema on the first day after keratoplasty was diagnosed as primary graft failure if other reasons for graft failure were excluded. [20].

### 2.3. Surgical Techniques

The surgical techniques of the first keratoplasty and each repeat keratoplasty were collected. The techniques involved in the current study were PKP, lamellar keratoplasty (LK), endothelial keratoplasty (EK), and keratolimbal allograft (KLAL). Anterior lamellar keratoplasty (ALK) and full-thickness deep anterior lamellar keratoplasty (DALK) are LKs. EK included deep lamellar endothelial keratoplasty (DLEK), Descemet stripping endothelial keratoplasty (DSEK), and Descemet membrane endothelial keratoplasty (DMEK).

### 2.4. Statistical Analysis

SPSS software (version 21; SPSS Inc, Chicago, IL) was used to perform the statistical analysis. The Chi-square test was applied to compare the differences in primary indications and surgical techniques among different groups and the distribution of reasons for regrafting. Pearson correlation coefficient and linear regression analyses were used to analyze the trend of the annual number of repeat keratoplasties. A *P* value less than 0.05 was considered significant.

## 3. Results

### 3.1. Demographic Data

**Figure 1:**
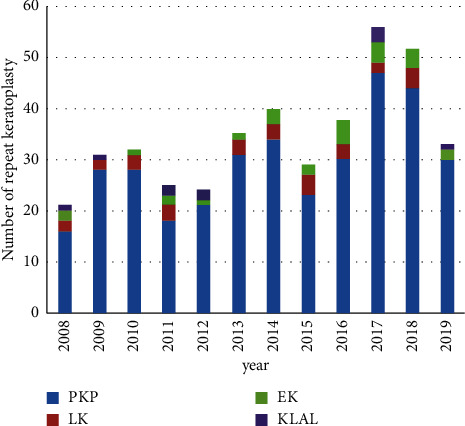
The annual number of repeat keratoplasties from 2008 to 2019. The total number of repeat corneal transplantations had a significant increasing trend in the study period.

A total of 418 eyes (411 patients, 275 men and 136 women) were included in the study. The mean age at repeat keratoplasty was 45.6 ± 20.9 years (range: 8 months ～87 years). Five hundred and thirty-one cases of repeat keratoplasty were performed during the study period, with a mean number of 34.8 ± 10.8 cases per year (range: 21–57). Nevertheless, the annual number of repeat keratoplasties increased by 2.6 times from 2008 to 2019, with the peak of 57 cases in 2017 and 52 cases in 2018. (Figure 1). Three hundred and thirty-seven eyes (80.6%) underwent only one repeat surgery. The median interval between the first and repeat keratoplasty was 25 months (range: 2 days to 57 years). Among the eyes that underwent multiple repeat keratoplasties, the numbers of eyes with the 3^rd^, 4^th^, 5^th^, 6^th^, and 7^th^ keratoplasty were 82, 22, 5, 3, and 1, respectively. The median interval between the two keratoplasties was 27.5 months (range: 1 month to 14 years). One hundred and twenty-four eyes (29.7%) had a history of concomitant ocular disease including cataract, glaucoma, ocular trauma, and vitreoretinopathy, and 90.3% of them (112 eyes) had a history of prior ocular surgery before the first keratoplasty. Compared to eyes with one regraft, eyes with multiple repeat keratoplasties were likely to have more concomitant ocular diseases and more prior surgeries. However, the differences were not significant (*P*=0.28 and *P*=0.14).

### 3.2. Primary Indications for Keratoplasty

The details of the primary indications for keratoplasty are listed in Table 1. A total of 256 eyes (61.2%) were classified as acquired nontraumatic among all cases, followed by congenital abnormalities (84 eyes, 20.1%) and acquired traumatic (78 eyes, 18.7%).

#### 3.2.1. Acquired Nontraumatic

Infectious keratitis was found to be the leading primary indication for repeat corneal transplantation in our study (99 cases, 23.7%). The majority of cases were caused by fungi (40 eyes, 40.4%) and viruses (36 eyes, 36.4%). Bullous keratopathy was the second common primary indication (62 eyes, 14.8%), which was predominantly caused by cataract surgeries (23 cases, 37.1%). Uncontrolled glaucoma (19 cases, 30.6%) was another common reason for bullous keratopathy, among which 18 eyes had a history of glaucoma surgery. Notably, although noninfectious keratitis was less common in this category (14 eyes, 3.4%), its proportion in multiple repeat keratoplasties (7 eyes, 8.6%) was significantly higher than that in single repeat keratoplasty (7 eyes, 2.1%, *P*=0.009).

#### 3.2.2. Congenital Abnormalities

This category included corneal dystrophy (42 eyes, 10.1%), congenital leucoma (27 eyes, 6.5%), and limbal dermoid (15 eyes, 3.6%). Compared with the other two categories, congenital abnormalities had a significantly lower proportion of multiple repeat keratoplasties than single repeat keratoplasty (*P*=0.025).

#### 3.2.3. Acquired Traumatic

Acquired traumatic included chemical injury (37 eyes, 8.9%), mechanical injury (30 eyes, 7.2%), and thermal injury (11 eyes, 2.6%). Notably, 37.8% of eyes with chemical injury underwent multiple repeat keratoplasties, which was significantly higher than other indications (*X*^2^ = 8.85, *P*=0.003).

#### 3.2.4. Comparison of Primary Indications between One Repeat Keratoplasty and Multiple Repeat Keratoplasties

Infectious keratitis (80 eyes, 23.7%) and bullous keratopathy (49 eyes, 14.5%) were the leading primary indications for one single repeat keratoplasty, while corneal degeneration such as band-shaped keratopathy and Terrien's marginal degeneration (6 eyes, 1.8%), and noninfectious keratitis (7 eyes, 2.1%) were the least common.

Among the eyes with multiple repeat keratoplasties, the second most common primary indication was chemical injury (14 eyes, 17.3%) rather than bullous keratopathy (13 eyes, 16%), although infectious keratitis remained the most common (19 eyes, 23.5%). Moreover, noninfectious keratitis was more common in this group than in one repeat keratoplasty group (8.6% vs. 2.1%, *P*=0.009). No cases of bacterial keratitis, amoeba keratitis, or limbal dermoid were found for multiple repeat keratoplasties.

### 3.3. Reasons for Repeat Corneal Transplantation

The leading reason for one repeat keratoplasty and multiple repeat keratoplasties was graft rejection (202 eyes, 48.3% and 60 eyes, 53.1%, respectively). The second most common reason was endothelial dysfunction (77 eyes, 18.4%) in eyes with one repeat keratoplasty and graft melting (20 eyes, 17.7%) in those with multiple repeat keratoplasties. Notably, 55 eyes (13.2%) with a stable ocular surface after the first keratoplasty underwent repeat keratoplasty for vision improvement because glycerol-preserved grafts were used in the first keratoplasty.

### 3.4. Surgical Techniques of Repeat Keratoplasty

**Figure 2:**
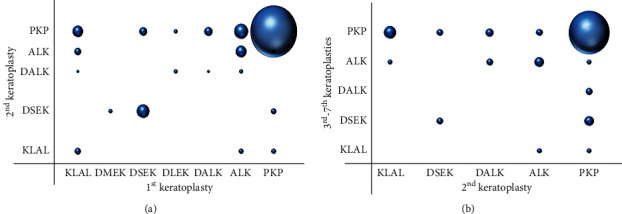
The distribution of surgical techniques in the first and repeat keratoplasties. (a) PKP-PKP (293 eyes, 70.1%), ALK-PKP (25 eyes, 6.0%), and DSEK-DSEK (21 eyes, 5.0%) were the most common procedures in the 1^st^ keratoplasty-2^nd^ keratoplasty group. (b) PKP-PKP (81 eyes, 71.7%) and KLAL-PKP (7 cases, 6.2%) were the main surgical techniques in 2^nd^ keratoplasty-3^rd^–7^th^ keratoplasty.

The surgical techniques used in the first keratoplasty and repeat keratoplasties are shown in Figure 2. PKP, ALK, and DSEK were mainly adopted. PKP accounted for 72.0% in the first keratoplasty, while its proportion significantly increased to 84.2% (447 cases) (*X*^2^ = 20.76, *P* < 0.001) in repeat keratoplasty, as shown in Table 3.

PKP was predominately used in both one repeat keratoplasty and multiple repeat keratoplasties. Among the eyes that underwent PKP in repeat keratoplasty, 293 eyes (83.2%) had PKP in the first keratoplasty. Similarly, among the eyes that underwent PKP in multiple repeat keratoplasties, 81 eyes (85.3%) received PKP in the prior surgery. Infectious keratitis (95 eyes, 22.7%) and corneal scarring (56 eyes, 13.4%) were the most common indications for PKP in the first repeat keratoplasty. Nevertheless, PKP was predominantly performed in multiple repeat keratoplasties to treat infectious keratitis (26 eyes, 23%) and chemical injury (14 eyes, 12.4%) (Table 4). Notably, 53 eyes underwent tectonic PKP in the first keratoplasty, and 52.8% of these eyes (28 eyes) underwent optical keratoplasty in the repeat surgery for vision improvement.

**Figure 3:**
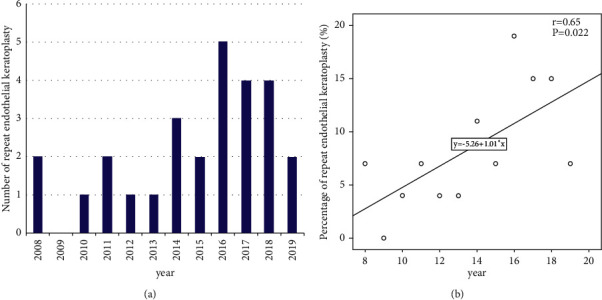
The annual number (a) of repeat endothelial keratoplasty procedures gradually increased from 2008 to 2019. Accordingly, its proportion in repeat keratoplasty (b) had a significant increasing trend.

EK has gradually become an alternative surgical technique for regrafting. Its proportion in repeat keratoplasty had a significant increasing trend from 2008 to 2019 (*r* = 0.65, *P*=0.022) (Figure 3). Nevertheless, DSEK was the only surgical procedure used in repeat EK in eastern China for the treatment of bullous keratopathy in one repeat keratoplasty (19 eyes, 4.5%) and corneal dystrophy in multiple repeat keratoplasties (4 eyes, 3.5%), which accounted for 72.4% in the treatment of failed endothelial keratoplasties.

The proportion of both ALK and KLAL decreased significantly in repeat keratoplasty compared to the first keratoplasty (*P*=0.005 and 0.001, respectively). KLAL was more frequently used in the treatment of chemical injury (11 eyes, 29.7%) as the first keratoplasty than the other indications (*P* < 0.01). However, in repeat keratoplasties, KLAL was not the preferential treatment for chemical injury (3 cases, 5.6%). Nevertheless, the proportion of DALK was similar in the first keratoplasty and repeat keratoplasty.

## 4. Discussion

According to previous studies, regrafting accounts for 23.1%∼40.9% of corneal transplantation in developed countries [1–5] and 5.3%∼24.5% in developing countries including China [8, 17, 21, 22]. Moreover, the absolute number of regrafts was reported to be 26.1∼53 cases per year in developed countries from 1989 to 2018 [1–5], which was almost ten times higher than that in China in the same period. [8, 17, 21, 22] Even in eastern China, a relatively developed area in China, the annual number of regrafting cases is still considerably low because of donor shortages [8, 17]. Nevertheless, the current study showed that the annual number of regrafting cases in eastern China increased gradually from 2008 to 2019 compared to a study published 15 years before [8]. With the establishment of a standardized national organ and tissue donating system, donor shortages might be alleviated, and the numbers of corneal transplantation and regrafting cases are expected to increase in the next decade.

The primary indications highly affect the outcomes of repeat keratoplasty [4, 7]. It has been confirmed that the severity and pathogen of infectious keratitis, the leading primary indication in the current study, was significantly correlated with its prognosis after PKP. [23, 24] Fungi and viruses are more likely to recur than other pathogens. Moreover, severe infectious keratitis usually has limbus and anterior chamber involved and requires larger-diameter grafts, which often leads to allograft rejection and failure. [25, 26] In addition, preoperative and postoperative inflammation and surgery-related complications also contribute to a high risk of graft failure, which makes regrafting necessary. [12, 24].

When fresh donors are unavailable, tectonic keratoplasty, which uses glycerol-preserved donor corneas in the majority of cases, is required to treat severe infectious keratitis and to reestablish the integral structure of the eyeball in a timely manner. A second-stage optical keratoplasty for visual rehabilitation is usually needed [27]. The present study showed that tectonic keratoplasty accounted for 53.5% and 25.3% of primary and repeat corneal transplantation, respectively, and most of them were PKP. However, its prognosis is far from satisfactory for various reasons [28]. It has been reported that therapeutic LK is effective in the treatment of advanced infectious keratitis, including fungal keratitis [28–30]. Compared with PKP, LK does not interfere with the anterior chamber and endothelium [23, 28, 29] and facilitates secondary optical keratoplasty. However, tectonic LK might have a high risk of infection recurrence because of possibly inadequate eradication of infectious tissue. A close follow-up after surgery and treatment will be helpful to improve the outcome [31].

Bullous keratopathy was also a common primary indication for repeat keratoplasty. It was reported that bullous keratopathy accounted for 36% of eyes having one repeat keratoplasty and 45% of cases with multiple repeat keratoplasties in developed countries [4, 6]. The major reason for bullous keratopathy in the current study was intraocular surgery, which destroyed anterior chamber-associated immune deviation and increased the risk of allograft rejection, endothelial decompensation and regrafting [32].

Severe chemical injury, the leading cause of total limbal stem cell deficiency in China [33], was one of the major indications for multiple repeat keratoplasties in the current study. Limbal stem cell transplantation is usually necessary to restore the function of limbal stem cells before PKP can be performed. As a result of technical limitations, allogeneic KLAL has been the only method of limbal stem cell transplantation used in eastern China in the past 12 years. Nevertheless, many reports have confirmed that immunosuppressive treatments are usually needed to help to maintain a stable ocular surface after KLAL, and repeat or even multiple KLAL are often required in severe cases of LSCD [34, 35]. The clinical application of cultivated epithelial transplantation and simple limbal epithelial transplantation provides more options for patients with chemical burns to reconstruct a stable ocular surface.

Notably, DSEK was the second most common surgical technique for repeat keratoplasty in the current study. Compared with PKP, EK has a smaller incision, fewer complications, and faster visual rehabilitation [12]. The technique of EK has been shifting from DLEK to DSEK and further to DMEK since its first clinical application in 2001 [36]. The efficacy and safety of DSEK as a technique to treat failed EK or even PKP has been confirmed. [12, 15, 37, 38] Although DMEK has been used as a method of repeat keratoplasty in developed countries since 2015 [39], its application in China is rare because of extreme donor shortages and difficulty in graft preparation.

The reasons for multiple repeat keratoplasties were not entirely the same as those for one repeat keratoplasty. Immune rejection and graft melting were the most common reasons for regrafting in multiple repeat keratoplasties. The current study showed that the proportion of eyes with graft melting after multiple repeat keratoplasties was almost ten times higher than that after the first keratoplasty. The primary indications that were inclined to cause graft melting were reported to be infection, autoimmune diseases and trauma [40, 41], which was consistent with our findings. Multiple repeat keratoplasties usually lead to a highly activated immune system and have a higher risk of infection, both of which possibly contribute to graft melting [41].

Two limitations should be addressed. First, the present study is a retrospective descriptive study based on medical charts. The outcomes after repeat keratoplasty, such as visual acuity and postoperative complications, were not available. Second, this is a single-center study. Although EENT hospital is the largest tertiary eye hospital and the first eye center that is qualified and authorized to perform keratoplasty in eastern China, selection bias might not have been avoided. Therefore, a multicenter, prospective study is needed to explore the relationship between indications, surgical techniques, and prognosis of repeat keratoplasty in the future.

In summary, infectious keratitis, bullous keratopathy, and chemical injury were the leading primary indications for repeat keratoplasty in eastern China from 2008 to 2019. Although PKP is predominantly used in repeat keratoplasty, the application of customized lamellar keratoplasty in regrafting is promising and might be more widely used in the treatment of graft failure in the near future. Thorough preoperative evaluation, cautious surgical selection and manipulation, close follow-up, and appropriate postoperative treatment are all crucial to improve the prognosis of patients after repeat keratoplasties.

## Figures and Tables

**Table 1:** The primary indications for keratoplasty.

Indications	Total	One repeat keratoplasty	Multiple repeat keratoplasties	*P*
Acquired nontraumatic	256 (61.2%)	204 (60.5%)	52 (64.2%)	0.54
Infectious keratitis	99 (23.7%)	80 (23.7%)	19 (23.5%)	0.96
Fungal keratitis	40 (40.4%)	33 (41.3%)	7 (36.8%)	0.75
Viral keratitis	36 (36.4%)	28 (35.0%)	8 (42.1%)	0.65
Bacterial keratitis	4 (4.0%)	4 (5.0%)	0 (0%)	—
Acanthamoeba keratitis	1 (1.0%)	1 (1.3%)	0 (0%)	—
Unknown	18 (18.2%)	14 (17.5%)	4 (21.1%)	1.0
Bullous keratopathy (except those caused by Fuchs dystrophy)	62 (14.8%)	49 (14.5%)	13 (16%)	0.73
Corneal scarring	56 (13.4%)	46 (13.7%)	10 (12.3%)	0.76
Keratoconus	15 (3.6%)	13 (3.9%)	2 (2.5%)	0.8
Noninfectious keratitis	14 (3.4%)	7 (2.1%)	7 (8.6%)	0.009
Corneal degeneration	7 (1.7%)	6 (1.8%)	1 (1.2%)	1
Others	3 (0.7%)	3 (0.9%)	0 (0%)	—
Congenital abnormalities	84 (20.1%)	75 (22.3%)	9 (11.1%)	0.025
Corneal dystrophy	42 (10.1%)	34 (10.1%)	8 (9.9%)	0.95
Congenital leucoma	27 (6.5%)	26 (7.7%)	1 (1.2%)	0.033
Limbal dermoid	15 (3.6%)	15 (4.5%)	0 (0%)	—
Acquired traumatic	78 (18.7%)	58 (17.2%)	20 (24.7%)	0.12
Chemical injury	37 (8.9%)	23 (6.8%)	14 (17.3%)	0.003
Mechanical injury	30 (7.2%)	27 (8.0%)	3 (3.7%)	0.18
Thermal injury	11 (2.6%)	8 (2.4%)	3 (3.7%)	0.78
Total	418 (100%)	337 (100.0%)	81 (100%)	—

*x* ^2^ test among the one repeat keratoplasty group and multiple repeat keratoplasties group.

**Table 2:** Reasons for repeat corneal transplantations.

Reasons	First repeat keratoplasties		Multiple repeat (3^rd^–7^th^) keratoplasties		*P*
Rejection	202	48.3%	60	53.1%	0.37
Endothelial dysfunction	77	18.4%	15	13.3%	0.2
For vision improvement	55	13.2%	5	4.4%	0.009
Recurrence of primary diseases	40	9.6%	7	6.2%	0.26
Graft infections	17	4.1%	2	1.8%	0.38
Graft melting	9	2.2%	20	17.7%	<0.001
Primary graft failure	4	1.0%	1	0.9%	1
Persistent epithelial defect	4	1.0%	0	0.0%	—
Graft dislocation	3	0.7%	0	0.0%	—
Trauma	2	0.5%	0	0.0%	—
Others	5	1.2%	3	2.7%	0.49
Total	418	100.0%	113	100.0%	

**Table 3:** The comparison on surgical techniques between the first keratoplasty and repeat keratoplasties.

	The first keratoplasty		Repeat (2^nd^–7^th^) keratoplasties		*P*
PKP	301	72.0%	447	84.2%	<0.001
LK	55	13.2%	39	7.3%	0.003
ALK	45	10.8%	31	5.8%	0.005
DALK	10	2.4%	8	1.5%	0.32
EK	35	8.4%	33	6.2%	0.2
DLEK	4	1.0%	0	0.0%	—
DSEK	29	6.9%	33	6.2%	0.65
DMEK	2	0.5%	0	0.0%	—
KLAL	27	6.5%	12	2.3%	0.001
Total	418	100.0%	531	100.0%	

PKP: penetrating keratoplasty; LK: lamellar keratoplasty; EK: endothelial keratoplasty; ALK: anterior lamellar keratoplasty; DALK: deep anterior lamellar keratoplasty; DLEK: deep lamellar endothelial keratoplasty; DSEK: Descemet stripping endothelial keratoplasty; DMEK: Descemet membrane endothelial keratoplasty; KLAL: keratolimbal allograft.

**Table 4:** The distribution of surgical indications of repeat keratoplasties.

Indications	One repeat keratoplasty					Multiple repeat keratoplasties				
	PKP	ALK	DALK	DSEK	KLAL	PKP	ALK	DALK	DSEK	KLAL
Acquired nontraumatic	217 (51.9%)	9 (2.2%)	5 (1.2%)	20 (4.8%)	5 (1.2%)	63 (55.8%)	7 (6.2%)	0	21 (1.8%)	1 (0.9%)
Infectious keratitis	95 (22.7%)	2 (0.5%)	1 (0.2%)	1 (0.2%)	0	26 (23%)	1 (0.9%)	0	0	0
Bullous keratopathy (except those caused by Fuchs dystrophy)	41 (9.8%)	0	2 (0.5%)	19 (4.5%)	0	13 (11.5%)	0	0	1 (0.9%)	0
Corneal scarring	56 (13.4%)	0	0	0	0	14 (12.4%)	0	0	1 (0.9%)	0
Keratoconus	13 (3.1%)	1 (0.2%)	1 (0.2%)	0	0	4 (3.5%)	0	0	0	0
Noninfectious keratitis	3 (0.7%)	5 (1.2%)	1 (0.2%)	0	5 (1.2%)	6 (5.3%)	5 (4.4%)	0	0	1 (0.9%)
Corneal degeneration	6 (1.4%)	1 (0.2%)	0	0	0	0	1 (0.9%)	0	0	0
Others	3 (0.7%)	0	0	0	0	0	0	0	0	0
Congenital abnormalities	64 (15.3%)	11 (2.6%)	0	7 (1.7%)	2 (0.5%)	11 (9.7%)	0	0	4 (3.5%)	0
Corneal dystrophy	34 (8.1%)	1 (0.2%)	0	7 (1.7%)	0	10 (8.8%)	0	0	4 (3.5%)	0
Congenital leucoma	27 (6.5%)	0	0	0	0	1 (0.9%)	0	0	0	0
Limbal dermoid	3 (0.7%)	10 (2.4%)	0	0	2	0	0	0	0	0
Acquired traumatic	71 (17.0%)	3 (0.7%)	1 (0.2%)	0	3 (0.7%)	21 (18.6%)	1 (0.9%)	2 (1.8%)	0	1 (0.9%)
Chemical injury	31 (7.4%)	2 (0.5%)	1 (0.2%)	0	3 (0.7%)	14 (12.4%)	1 (0.9%)	2 (1.8%)	0	0
Mechanical injury	30 (7.2%)	0	0	0	0	3 (2.7%)	0	0	0	0
Thermal injury	10 (2.4%)	1 (0.2%)	0	0	0	4 (3.5%)	0	0	0	1 (0.9%)
Subtotal	352 (84.2%)	23 (5.5%)	6 (1.4%)	27 (6.5%)	10 (2.4%)	95 (84.1%)	8 (7.1%)	2 (1.8%)	6 (5.3%)	2 (1.8%)
Total	418 (100%)					113 (100%)				

PKP: penetrating keratoplasty; ALK: anterior lamellar keratoplasty; DALK: deep anterior lamellar keratoplasty; DSEK: Descemet stripping endothelial keratoplasty; KLAL: keratolimbal allograft.

## Data Availability

The data used to support the findings of this study are included within the article.
